# Endoplasmic Reticulum Stress and Parkinson's Disease: The Role of HRD1 in Averting Apoptosis in Neurodegenerative Disease

**DOI:** 10.1155/2013/239854

**Published:** 2013-04-18

**Authors:** Tomohiro Omura, Masayuki Kaneko, Yasunobu Okuma, Kazuo Matsubara, Yasuyuki Nomura

**Affiliations:** ^1^Department of Clinical Pharmacology and Therapeutics, Kyoto University Hospital, Sakyo-ku, Kyoto 606-8507, Japan; ^2^Laboratory of Medical Therapeutics and Molecular Therapeutics, Gifu Pharmaceutical University, 1-25-4 Daigaku-Nishi, Gifu 501-1196, Japan; ^3^Department of Pharmacology, Faculty of Pharmaceutical Sciences, Chiba Institute of Science, Choshi, Chiba 288-0025, Japan; ^4^Laboratory of Pharmacotherapeutics, Yokohama College of Pharmacy, Yokohama 245-0066, Japan

## Abstract

Endoplasmic reticulum (ER) stress has been known to be involved in the pathogenesis of various diseases, particularly neurodegenerative disorders such as Parkinson's disease (PD). We previously identified the human ubiquitin ligase HRD1 that is associated with protection against ER stress and its associated apoptosis. HRD1 promotes the ubiquitination and degradation of Parkin-associated endothelin receptor-like receptor (Pael-R), an ER stress inducer and causative factor of familial PD, thereby preventing Pael-R-induced neuronal cell death. Moreover, upregulation of HRD1 by the antiepileptic drug zonisamide suppresses 6-hydroxydopamine-induced neuronal cell death. We review recent progress in the studies on the mechanism of ER stress-induced neuronal death related to PD, particularly focusing on the involvement of HRD1 in the prevention of neuronal death as well as a potential therapeutic approach for PD based on the upregulation of HRD1.

## 1. Introduction

The endoplasmic reticulum (ER), an organelle found in the cells of eukaryotes, plays a key role in protein synthesis, glycosylation, and folding [1]. ER stress caused by glucose starvation, hypoxia, disruption of calcium homeostasis, or oxidative stress leads to the accumulation of unfolded or misfolded proteins. This induces cellular physiologic protective responses termed as the unfolded protein response (UPR). However, during prolonged ER stress, unfolded proteins may stimulate specific proapoptotic pathways through the activation of the transcription factor C/EBP homologous protein (CHOP) and cysteine proteases caspase-4/12 [2–6].

**Figure 1:**
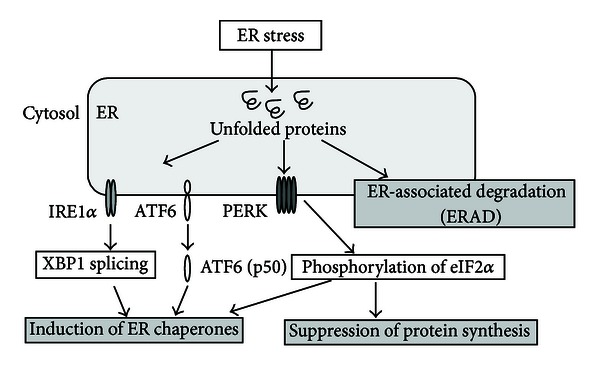
Cellular responses to ER stress. ER stress due to hypoxia and other factors results in the accumulation of unfolded proteins that trigger the UPR. The UPR is composed of three pathways: induction of ER chaperones by the activation of IRE1*α*-XBP1, ATF6, and PERK-eIF2*α*, inhibition of protein synthesis by the phosphorylation of PERK-eIF2*α*, and ERAD.

The UPR includes the repression of protein synthesis via phosphorylation of the *α* subunit of the eukaryotic initiation factor 2*α* (eIF2*α*), which is promoted by the activation of protein kinase RNA-like ER kinase (PERK) [7], and degradation of the unfolded proteins by ER-associated degradation (ERAD) (Figure 1). An additional key UPR pathway is the promotion of appropriate protein folding through induction of ER chaperones via activation of the activating transcription factor 6 (ATF6), PERK, and inositol-requiring enzyme-1 (IRE1) (Figure 1) [8]. ATF6 is cleaved by proteases under ER stress, yielding an active cytosolic ATF6 fragment p50 that migrates to the nucleus and activates ER chaperones [9]. PERK dimerization and transautophosphorylation lead to the phosphorylation of eIF2*α*, reducing the overall frequency of mRNA translation initiation. However, ATF4 mRNA is preferentially translated in the presence of phosphorylated eIF2*α*. ATF4 activates the transcription of ER chaperones [10, 11]. Furthermore, autophosphorylation and oligomerization of IRE1 activate IRE1 endoribonuclease, resulting in X-box binding protein 1 (XBP1) mRNA cleavage and splicing. The transcription factor XBP1 regulates genes responsible for ERAD, as well as genes associated with protein folding [12].

**Figure 2:**
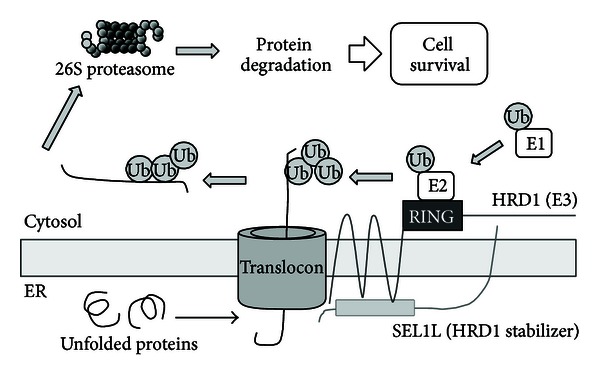
Mechanism of the ERAD system. Unfolded proteins are retrotranslocated from the ER to the cytosol through the translocon. Substrates are then polyubiquitinated by E1, E2, E3, and other components and are subsequently degraded by the 26S proteasome, resulting in cell survival. HRD1 and SEL1L are components of the ERAD system that colocalize in the ER and interact with one another. HRD1 has E3 activity, and SEL1L regulates E3 activity and HRD1 stability.

During ERAD, unfolded proteins are retrotranslocated to the cytosol from the ER via the translocon; polyubiquitinated by the ubiquitin-activating enzyme (E1), ubiquitin conjugating enzyme (E2), ubiquitin ligase E3, and other components; and finally degraded by the 26S proteasome (Figure 2). The RING finger domain of E3 plays a particularly important role in the ubiquitination of unfolded proteins, mediating the transfer of ubiquitin from E2 to substrates [13, 14]. 

ER stress has been proposed as a possible molecular mechanism underlying the onset of diabetes mellitus [15–17], rheumatoid arthritis [18, 19], and neurodegenerative diseases such as Parkinson's disease (PD) [20–23] and Alzheimer's disease (AD) [24]. This discovery of a link between ER stress and disease onset indicates that unfolded proteins play a role in the etiology of many of the most prevalent diseases. It has been suggested that therapeutic drug targeting and other interventions aimed at disrupting the ER stress cycle in such diseases would provide a useful treatment strategy [25–27].

Here we review recent evidence for the involvement of ER stress in PD. Furthermore, we describe the suppressive roles of the ubiquitin ligase HRD1 in ER stress-induced neuronal death and propose a new approach for the treatment of PD focusing on HRD1.

## 2. PD and ER Stress

PD is the most common movement disorder, particularly in the elderly, and is the second most common neurodegenerative disease. It is characterized by motor symptoms including bradykinesia, rigidity, resting tremor, and postural instability. The pathological hallmark of PD is the loss of dopaminergic neurons in the substantia nigra pars compacta (SNC), resulting in a reduction of the dopamine content in the striatum [28, 29].

Cases of PD are mostly sporadic, and it is estimated that only approximately 5–10% of patients exhibit monogenic forms of the disease [30]. The genes involved in autosomal recessive PD are *Parkin* (*PARK2*), *PTEN-induced putative kinase 1* (*PINK1*; *PARK6*), *DJ-1* (*PARK7*), and *ATP13A2* (*PARK9*), whereas the genes involved in autosomal dominant PD are *α-synuclein* (*PARK1/4*), *leucine-rich repeat kinase 2* (*LRRK2*; *PARK8*), and *ubiquitin carboxy-terminal hydrolase L1* (*UCHL-1*; *PARK5*) [31]. In addition, a genome-wide association study in individuals of Japanese and European ancestries found that *LRRK2* and *α-synuclein* are common risk factors for sporadic PD [32, 33]. LRRK2 and *α*-synuclein are substrates for ERAD-related E3, C-terminus of Hsp70-interacting protein (CHIP), and Parkin, demonstrating that disturbances in the ERAD system are relevant to the onset of PD [34–37]. Based on these reports, it is presumed that ER stress is a causative factor of PD.

Parkin is an E3 containing two RING finger motifs that bind one or more ubiquitin molecules, thereby targeting the substrate for proteasomal degradation [37]. Through its E3 activity, Parkin degrades its own substrate, the misfolded Parkin-associated endothelin receptor-like receptor (Pael-R) and, thus, suppresses cell death caused by the accumulation of Pael-R [20]. However, Parkin mutation results in the loss of E3 activity, which can cause the accumulation of unfolded Pael-R and finally ER stress-induced cell death [34]. Therefore, ER stress that occurs as a result of the accumulation of unfolded Pael-R is suggested to be one of the pathophysiological mechanisms underlying autosomal recessive PD [38, 39].

Pael-R also accumulates in the core of Lewy bodies in sporadic PD [40]. However, Parkin-deficient mice exhibit no significant changes in either dopaminergic neurodegeneration or in the accumulation of any Parkin substrates [41–43]; this contrasts with the results of a study showing that Parkin knockout/Pael-R transgenic mice exhibit progressive loss of dopaminergic neurons [44]. These reports suggest that other E3s are capable of degrading accumulated Pael-R in the absence of Parkin, as a compensatory mechanism for the maintenance of cellular homeostasis.

Two recent studies highlight the roles of PINK1 and Parkin in PD. PINK1 and Parkin work together to regulate mitochondrial fission [45, 46]. In particular, autophosphorylation of PINK1 reportedly recruits Parkin to damaged mitochondria and Parkin then initiates the mitochondrial degradation; however, these events are averted in PD by mutations of PINK1 [47, 48]. These key findings demonstrate the importance of E3 Parkin in the onset of PD.

## 3. HRD1 and PD

We previously identified HRD1, a human homolog of yeast Hrd1p/Der3p [49]. Hrd1p/Der3p is a RING finger domain-containing E3 localized to the ER and is involved in ERAD and ubiquitination of HMG-CoA reductase (Hmg2p) [50, 51]. We also identified the HRD1-stabilizer SEL1L, a human homolog of Hrd3p [52, 53]. We demonstrated that HRD1 has E3 activity, mRNA and protein levels of HRD1 are upregulated in response to ER stress, and HRD1 inhibits ER stress-induced cell death [53]. Furthermore, it has been reported that HRD1-SEL1L complex components OS-9 and GRP94 are responsible for delivering substrate [54], indicating that HRD1 ubiquitinates substrates cooperatively with SEL1L, OS-9, and various other ERAD-related components; it is believed that Derlin-1, XTP3-B, or other molecules may also play similar delivery roles in the complex [54, 55], but detailed functional analysis of such molecules remains unclear. Our studies demonstrated that HRD1 expression is reduced by the knockdown of SEL1L [49].

Based on the above-mentioned findings, it is presumed that overexpressed HRD1 degrades many unfolded proteins, resulting in the inhibition of cell death caused by ER stress. Thus, we searched for endogenous HRD1 substrates related to ER stress and focused on Pael-R, because of its importance in causing ER stress [56].

**Figure 3:**
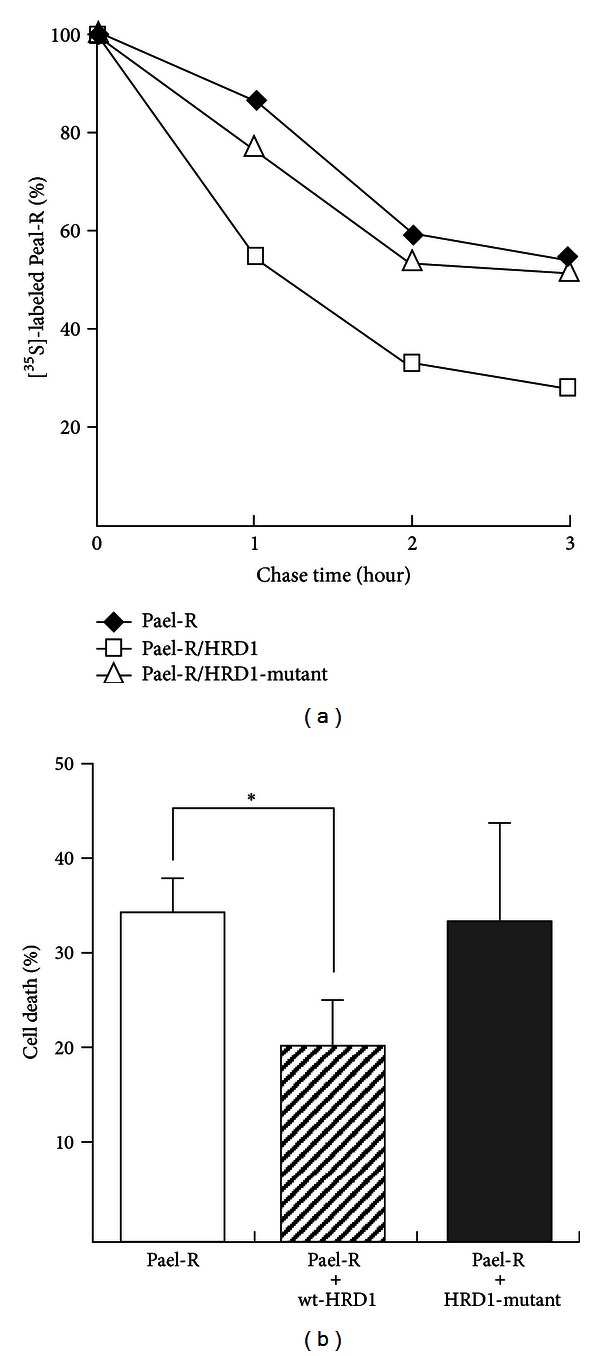
HRD1 degrades unfolded Pael-R and suppresses Pael-R-induced cell death. (a) Neuro2a cells were transiently transfected with Pael-R and HRD1, or HRD1-mutant. At 36 h after transfection, cells were pulse-labeled with [^35^S]-methionine/cysteine and chased for the indicated times. The levels of  [^35^S]-labeled Pael-R are plotted relative to the amount present at time 0. (b) HEK293 cells (control) and HEK293 cells expressing HRD1 or HRD1-mutant were transiently transfected with Pael-R. The surviving cells were stained with crystal violet. The percentage of cell death was calculated as follows: 100 – ((optical density for assay/optical density for control well) × 100). The results obtained from each cell transfected with Pael-R were compared with those obtained from cells transfected with control vector. The results are expressed as the means ± S.D. of three independent experiments performed in duplicate. Statistical analysis was performed using Student's *t-*test (*P < 0.01 versus normal) [56].

Because human HRD1 has been reported to be expressed in the brain by RT-PCR—ELISA studies [57], we examined the localization of HRD1 in the murine brain and demonstrated that it is expressed in the SNC, particularly in dopaminergic neurons [56]. It has been reported that Pael-R is also expressed in SNC dopaminergic neurons [20]. Thus, we hypothesized and demonstrated that HRD1 and Pael-R exist in correlation with one another; HRD1 and Pael-R colocalize in the ER in dopaminergic SH-SY5Y cells, and HRD1 interacts with unfolded Pael-R. Furthermore, we demonstrated that Pael-R is ubiquitinated and degraded by HRD1 and that Pael-R-induced cell death is suppressed by the overexpression of HRD1 (Figure 3).

Moreover, we demonstrated that HRD1 is expressed in neurons, but not in glial cells, of the murine brain, and that this ligase is also expressed in the pyramidal cell layer of the hippocampus, globus pallidus, striatum, and Purkinje cells of the cerebellar cortex, in addition to the SNC dopaminergic neurons. It has been reported that these regions are injured in various neurodegenerative disorders, particularly in motor dysfunctions such as PD, Huntington's disease, spinocerebellar ataxia, and prion diseases [28, 29, 58–60]. Therefore, it is plausible that HRD1 may be associated with the onset of other motor dysfunctions. 

A detailed functional analysis revealed that in addition to the RING finger domain, HRD1 contains a proline-rich domain involved in interaction with Pael-R, as well as a transmembrane domain [56, 61]. The transmembrane domain of HRD1 transports Pael-R from the ER to the cytosol and is also needed to stabilize HRD1 itself [61]. We previously reported that SEL1L stabilizes HRD1 [49], and, more recently, Fonseca et al. [62] reported Wolfram syndrome 1 protein as another HRD1 stabilizer. Therefore, as HRD1 was not able to interact with SEL1L or other components without the transmembrane domain, we assume that HRD1 had lost its stability.

## 4. Treatment Strategies for PD Involving ER Stress

**Figure 4:**
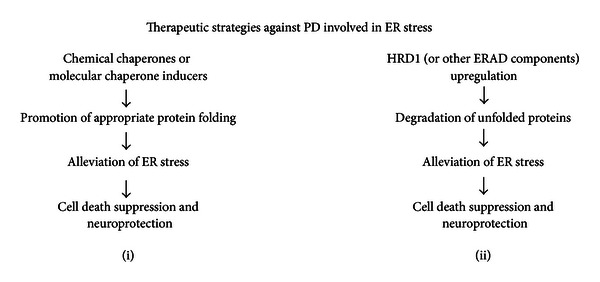
Therapeutic strategies for PD involving ER stress. (i) Addition of chemical chaperones (e.g., 4-PBA and tauroursodeoxycholic acid) or molecular inducers of ER chaperones (e.g., BIX); these molecules promote the appropriate folding of proteins and suppress the accumulation of unfolded proteins and ER stress-induced cell death, resulting in the prevention of neurodegeneration in PD, and (ii) the upregulation of ubiquitin ligase HRD1, its stabilizer SEL1L, or other ERAD components; HRD1 and its components promote the degradation of unfolded proteins and suppress ER stress-induced cell death, resulting in the prevention of neurodegeneration in PD.

Based on the above-mentioned findings, we propose the following therapeutic strategies for PD involving ER stress: (i) the promotion of appropriate protein folding to avoid ER stress or (ii) the upregulation of HRD1 or its related components to promote the degradation of unfolded proteins (Figure 4). With respect to (i), it has been reported that chemical chaperones or molecular chaperone inducers promote the appropriate folding of proteins [63–65]. We similarly reported that the chemical chaperone 4-phenyl butyrate (4-PBA) or its derivatives promote the correct folding of unfolded Pael-R and suppress the cell death caused by the accumulation of Pael-R [66, 67]. In addition, 4-PBA improves motor deterioration in human *α*-synuclein A30P/A53T double-transgenic mice [68] and prevents memory deficits and decreases amyloid *β* in AD transgenic mice [69]. Furthermore, the molecular chaperone inducer Bip inducer X (BIX) prevents ER stress-induced neuronal death [63]. Based on these reports, the acceleration of appropriate protein folding using chemical chaperones or BIX is considered to be useful for the treatment of PD and other neurodegenerative disorders caused by ER stress.

Regarding treatment strategy (ii), we have been trying to identify chemicals that promote the expression of HRD1 proteins; through this research, we identified the antiepileptic drug zonisamide as an upregulator of HRD1 [70]. Zonisamide has recently been shown to improve the cardinal symptoms of PD and is approved in Japan for use as a low-dose adjunctive therapy for PD patients [71, 72]. However, the molecular mechanisms through which zonisamide suppresses the progression of PD remain unclear. We have demonstrated that a low concentration of zonisamide suppresses neuronal cell death caused by 6-hydroxydopamine-induced ER stress [70]. Zonisamide upregulates the HRD1 protein, without upregulating HRD1 mRNA, through a mechanism involving SEL1L. It upregulates expression of SEL1L mRNA and protein, resulting in the stabilization of HRD1 protein and followed by an increase in the HRD1 protein level. In contrast, knockdown of SEL1L downregulates HRD1 and suppresses the protective effect of zonisamide against ER stress. These findings indicate that zonisamide may activate SEL1L to act as an HRD1 stabilizer, and the resulting upregulated HRD1 proteins repress 6-hydroxydopamine-induced cell death [70].

## 5. Conclusions

We reviewed the involvement of ER stress in the etiology of PD, the critical role of HRD1 as a ubiquitin ligase in ERAD, and a therapeutic strategy against PD based on HRD1. PD has recently been reported to be a multifactorial neurodegenerative disease; therefore, it is important to approach its treatment from different angles, including environmental factors, oxidative stress, and mitochondrial dysfunction, in addition to ER stress [73–75]. We also described important findings demonstrating the involvement of HRD1 in the degradation of the amyloid precursor protein and the subsequent reduction of amyloid *β*, a possible factor in the pathogenesis of AD [24].

Based on these findings, we propose that HRD1 has a variety of substrates underlying protein conformational diseases, including PD and AD, and speculate that the molecules that activate HRD1 may have therapeutic potential for the treatment of neurodegenerative disorders. If ER stress is indeed one of the causes of neurodegenerative diseases, it is possible that this approach represents a common neuroprotective strategy that can be exploited for the treatment of neurodegenerative disorders in general.
